# Hospital biopreparedness in the Looming Presence of SARS‐CoV‐2/COVID‐19

**DOI:** 10.1002/hsr2.149

**Published:** 2020-03-09

**Authors:** Saskia Popescu

**Affiliations:** ^1^ School of Public Policy, Biodefense George Mason University Fairfax Virginia USA; ^2^ HonorHealth Phoenix Arizona USA

1

According to the World Health Organization, as of February 23, 2020, there have been over 78 000 confirmed cases of the novel coronavirus that first caused illness in Wuhan, China: the SARS‐CoV‐2 virus. As quarantines and travel bans are put into place—frustrating public health experts—there is an increasing need to address the hospital dynamics of response to such biological events.

The recent analysis of 138 hospitalized patients in Wuhan, China, and their clinical characteristics, has given credence to stronger investments into hospital biopreparedness and overall infection prevention efforts.^1^ Within this analysis, researchers found two pieces that are particularly relevant to healthcare preparedness: first, that 26% of patients required admission to an intensive care unit, and second, that 41% of cases were related to healthcare transmission. Patients requiring medical care in an intensive care unit inherently burden the system more, both in terms of supplies in personnel, but also because they typically have greater lengths of stay. The volume of healthcare‐associated cases is an indicator of infection prevention breakdowns, which points to the potential for hospitals to further spread the disease.

This is not a unique finding, however, and similar situations have been observed in previous coronavirus outbreaks. Indeed, hospitals can easily act as amplifiers for disease transmission during these events. In 2003, the Severe Acute Respiratory Syndrome coronavirus (SARS‐CoV) outbreak in Toronto highlighted this very real vulnerability, where busy emergency departments, delays in isolation, and improper personal protective equipment (PPE) use fueled the spread of disease in several hospitals.^2^ Enhanced infection prevention measures were eventually implemented (higher level of PPE, masking when in public areas, among others), which helped bring transmission to halt. After the outbreak was believed to be over, however, directives were given to discontinue those enhanced infection prevention measures. As a result of this and staff no longer routinely wearing masks in general hospital areas, a second phase of the outbreak began. An overwhelming majority of cases in Phase II of the Toronto SARS‐CoV were related to healthcare transmission.^3^

A 2015 outbreak of Middle East Respiratory Syndrome coronavirus (MERS‐CoV) also highlighted the ability for hospitals and breaks in infection prevention to help fuel coronavirus transmission. A single patient in an overcrowded emergency department helped propel the virus.^4^ Further analysis would reveal that multi‐patient rooms, the expectation that family partake in the care process, and hospital‐shopping all helped the disease spread.^5^ Of the 186 cases confirmed, 184 were a result of nosocomial transmission, and as Kim et al noted, it was “largely attributable to infection management and policy failures rather than biomedical factors.”^5^

The propensity for infectious diseases, especially novel or emerging, to spread throughout healthcare facilities should not be ignored in this current outbreak. As the world struggles with supplies of PPE, efforts should be made to focus on the full spectrum of infection prevention efforts. Hospital biopreparedness requires a holistic effort that includes reinforcement of isolation precautions, hand hygiene, environmental disinfection, and surge planning. Adequate staffing, consideration for an influx of patients, properly working airborne isolation rooms (AIIR), and engaged hospital leadership will all be necessary as hospitals everywhere work to prepare for potential cases.

It is critical to note, though, that the infection prevention requirements for SARS‐CoV‐2/COVID‐19 are not a new strategy for healthcare workers. Airborne and contact isolation precautions are well‐established strategies that healthcare workers already employ, meaning that unlike the requirements for Ebola virus disease preparedness in 2014, healthcare workers already have the skills and capabilities to protect themselves and patients. Employment of the i3 strategy (identify, isolate, and inform) and those foundational infection prevention practices are necessary to help avoid additional cases of COVID‐19, in addition to other infectious diseases.

Healthcare investment in biopreparedness is often intermittent and frequently provided only in the face of an outbreak or biological event. This current outbreak should be seen as an opportunity to ensure those efforts are being supported and reinforced by leadership. Hospital leaders often note that investment in prevention and response efforts for seemingly improbable biological events is unlikely due to competing interests.^6^ Healthcare biopreparedness goes beyond just ensuring we have enough PPE, but also requires instilling the infection prevention foundation that patient and employee safety precariously rests upon. The future should see international efforts to strengthen the critical healthcare and public health infrastructure across the world, as these vulnerabilities are systemic and deserve the highest attention.
